# Nurses’ experiences of providing medical services during the Kermanshah earthquake in Iran: a qualitative study

**DOI:** 10.1186/s12873-023-00920-9

**Published:** 2024-01-07

**Authors:** Khadijeh Akbari, Arezoo Yari, Abbas Ostadtaghizadeh

**Affiliations:** 1 Department of Health in Emergencies and Disasters, School of Public Health, Tehran University of Medical Sciences, Tehran, Iran; 2 Department of Nursing, 501 Hospital (Imam Reza), AJA University of Medical Sciences, Tehran, Iran; 3 Social Determinants of Health Research Center, Research Institute for Health Development, Kurdistan University of Medical Sciences, Sanandaj, Iran; 4 Department of Health in Emergencies and Disasters, School of Medicine, Kurdistan University of Medical Sciences, Sanandaj, Iran

**Keywords:** Nursing experiences, Natural Disasters, Earthquakes, Kermanshah, Iran

## Abstract

**Background:**

Nurses have always been at the forefront of providing services for victims of disasters. Using nurses’ experiences in disaster planning can play an important role in improving their readiness to provide healthcare during disasters. The objective of this study is not only to understand the challenges but also to explore and document the broader spectrum of experiences encountered by nurses in these critical situations. By focusing on their experiences, we aim to contribute valuable insights to enhance disaster preparedness and healthcare delivery strategies.

**Methods:**

This qualitative study employed the content analysis method to describe the experiences of 16 earthquake relief nurses in Kermanshah, Iran. Sampling was done purposefully and continued until data saturation was achieved. Initially, two unstructured interviews were conducted to shape the interview’s main line and refine guide questions. Subsequently, the study involved semi-structured interviews and observation notes for a nuanced understanding of the participants’ experiences.

**Results:**

In the analysis of the interviews, 920 codes were obtained and the nurses’ experiences were categorized into three main categories: personal experiences, operational experiences, and social and cultural experiences. These three categories covered 12 subcategories.

**Conclusion:**

Results of describing nurses’ experiences in the Kermanshah earthquake showed that nurses need to plan and implement necessary measures to ensure pre-disaster preparedness to respond effectively to disasters such as earthquakes. Besides, it is necessary to prepare, train and practice these interventions regularly, periodically, and purposefully. They should be evaluated and updated if they are used in a real earthquake or practice and maneuver.

**Supplementary Information:**

The online version contains supplementary material available at 10.1186/s12873-023-00920-9.

## Background

Over the past decades, there has been an increasing trend in the number of disasters worldwide. Meanwhile, earthquakes have always been in the first ranks of the natural hazards induced disasters list, to the extent that they are considered the most important deadly disasters in the world in recent years. The statistics recorded in the Emergency Events Database (EM-DAT) at the Centre for Research on the Epidemiology of Disasters (CRED) also confirm such growth and 792 earthquakes were reported between 1987 and 2015 [1].

In fact, 80% of earthquake-related casualties have occurred in only six countries, including Iran. The Iranian plateau is one of the most seismically active regions in the world, and now and then, a destructive and disastrous earthquake occurs with extensive human and financial damage. For example, we can refer to the earthquakes of Salmas (1930), Buin Zahra (1962), Tabas (1978), Rudbar-Manjil (1990), Bam (2003), Varzaghan (2012) and Kermanshah (2017) [2].

The Kermanshah earthquake, with a magnitude of 7.3, occurred on the evening of November 21, 2017, near Azgale in the Kermanshah province. The epicenter was located near the border of Iran and Iraq, approximately 32 km southwest of Halabcheh, Iraq. According to statistics, 620 people were killed, 8000 people were injured, about 70,000 people became homeless, 4,700,000 were affected, and more than 12,000 buildings were damaged in this earthquake [3]. Some health centers and government buildings, including the crisis management building, were seriously damaged or destroyed, and due to the destruction of roads and the collapse of the mountain, relief was limited in some places [4]. In contrast to previous seismic events, the current earthquake witnessed a heightened mobilization of support for victims at local, national, and global levels, attributable to the influential role of social media.[5], but due to the preference of the people and weakness government institutions, self-centered actions in the distribution of goods and the creation of heavy traffic made it difficult to provide relief at the beginning of the earthquake [4].

Considering the health consequences of disasters, especially earthquakes, the health system is special in providing health services before, during, and after such events. Accordingly, the proper preparation of healthcare providers is essential [6]. Nurses comprise the largest group of healthcare providers who play an important role in disaster response [7]. Although nurses should be at the forefront of health service providers in the response phase, they may feel unprepared due to limited resources [8] to the extent that they always face many problems and challenges when fulfilling their role at the time of disasters [9]. When providing services to earthquake victims, they face the following problems: difficult and stressful work conditions, unfavorable teamwork conditions, feeling incompetent [10], lack of physical and mental preparation during the deployment process, an inadequate pre-deployment program and training [8], ethical challenges [11]. Moreover, nurses should be able to provide care methods according to the conditions and be aware of their role to respond effectively in the face of a disaster. This preparation is achieved through relevant training and acquisition of practical skills, managerial experiences, understanding of capabilities, and local and regional resources [12].

According to previous studies, nurses should have the following five most important abilities and necessary professional qualifications during disasters: managerial, ethical, and legal qualifications, specific technical qualifications, and teamwork and personal abilities [13]. Studies also revealed the effectiveness of disaster training courses and exercises in improving the nurses’ knowledge, skills, and preparedness for disasters [6, 8]. National guidelines and concepts for disaster nursing should be developed, and nurses should be aware of their duties [14].

People’s risk perception is widely acknowledged as a critical factor in responding to disasters [15]. Recognizing that individuals’ risk perception is shaped by their experiences [16], the unique experiences derived from nurses’ encounters with disasters play a pivotal role in enhancing overall risk perception. Consequently, these experiences contribute significantly to effective disaster management. In fact, such experiences will improve nurses’ preparedness in the face of disasters [17]. Although Iran is one of the most disaster-prone countries [2]. However, studies about the position of nurses in the disaster team and their skills and competence in responding to disasters are limited [13].

Planning and preparing nurses for effective disaster management constitute fundamental principles in the realm of healthcare during disasters. Given the seismicity of Iran and the pivotal role of nurses in delivering healthcare services during the response phase, it is imperative for health managers to intricately consider nurses’ experiences in their planning endeavors. In line with this, our study aims not only to understand the challenges but also to comprehensively explore and meticulously document the full spectrum of experiences encountered by nurses in these critical situations. By focusing on their firsthand experiences, our goal is to provide valuable insights into the nuanced realities faced by nurses. Through this exploration, we aspire to contribute to the broader discourse on disaster preparedness and healthcare delivery strategies, aiming to enhance the resilience and effectiveness of our healthcare systems in the face of unforeseen adversities.

## Materials and methods

In this research, a qualitative research design and the conventional content analysis method were utilized to elucidate the experiences of nurses involved in earthquake relief efforts in Kermanshah, Iran. Content analysis is a systematic method that aims to describe the depth and breadth of a phenomenon, leading to the revision of information, valid inferences, and the production of new insights and knowledge. It is particularly suitable for investigating people’s experiences and attitudes towards a particular topic and focuses on individuals’ life experiences, interpretations, and meanings [18].

Therefore, in this study, the conventional content analysis method was utilized to identify explicit and implicit concepts based on the descriptions provided by the study participants.

### Participants and setting

**Fig. 1:**
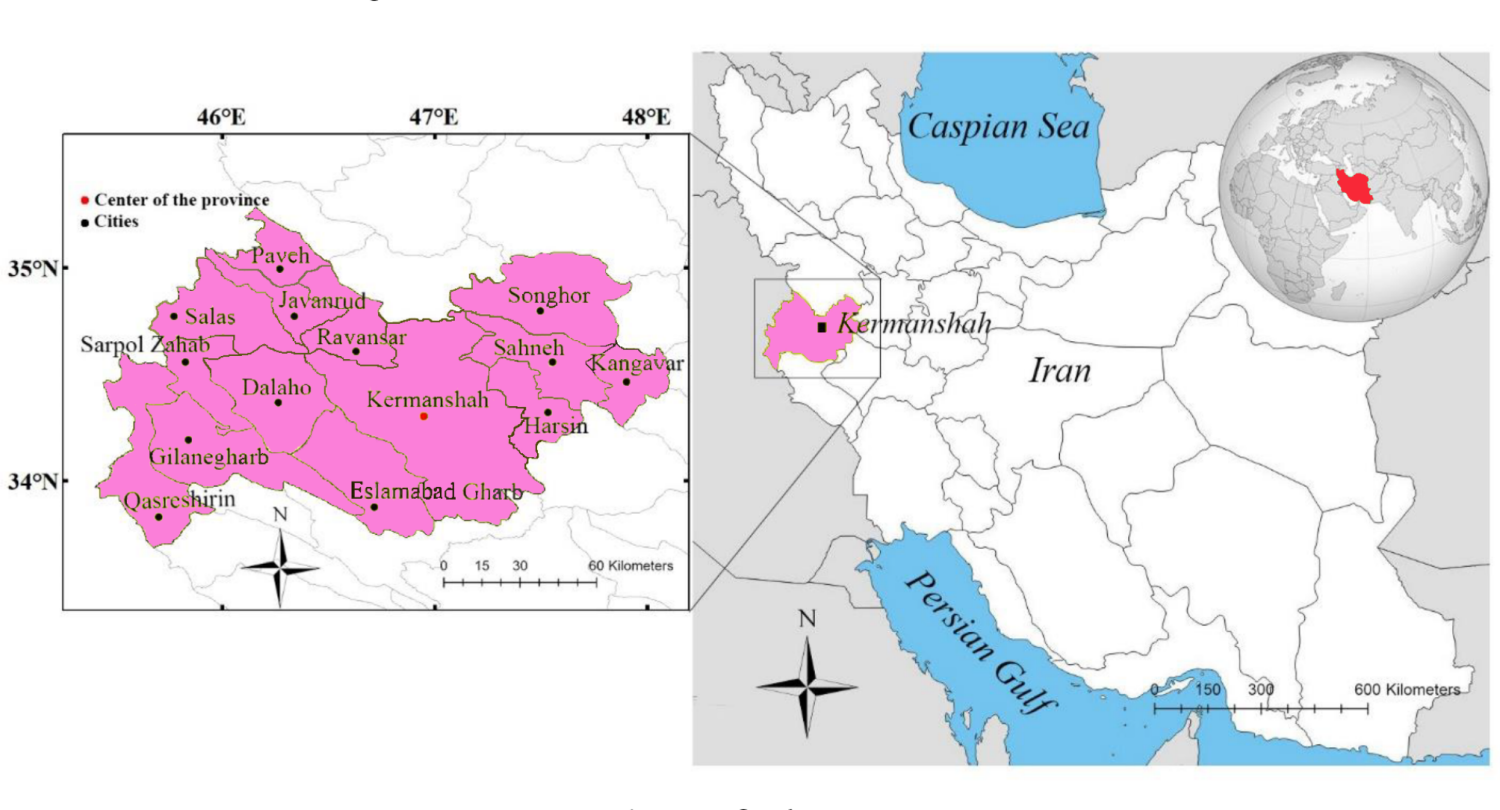
This map was reformed by the authors using existing data the following reference: Rezapour S, Jooyandeh E, Ramezanzade M, Mostafaeipour A, Jahangiri M, Issakhov A, et al. Forecasting rainfed agricultural production in arid and semi-arid lands using learning machine methods: A case study. Sustainability. 2021; 13(9):4607

The participants included nurses in the earthquake-affected areas of Kermanshah Province to provide aid services and were dispatched to these areas under the supervision of the Tehran University of Medical Sciences affiliated with the Ministry of Health and Medical Education of Iran (Fig. 1). The names of the participants were taken from the Tehran University of Medical Sciences. Coordination with them involved contacting by phone to assess their willingness to participate in the research.

This study initially employed convenience sampling to facilitate the commencement of interviews. However, as indicated in Table 1, after the second or third interview, we transitioned to a purposive sampling approach, specifically employing maximum variation based on factors such as sex, marital status, years of work experience, and presence during the Kermanshah earthquake. The shift to purposive sampling aimed to ensure a diverse and comprehensive representation of participants with varying characteristics related to the research topic. The decision to combine convenience and purposive sampling methods was made to balance practicality and efficiency in quickly gathering data from a broad pool of potential participants while also ensuring the inclusion of individuals with specific and relevant experiences.

**Table 1:** Demographic characteristics of study participants

Demographic characteristics	Number (%)
Gender	
Female Male	9 (56, 25) 7 (43, 75)
Age (years)	
25–34 35–44 45 and above	5 (31, 25) 8 (50) 3 (18, 75)
Marital status	
Married Single	10 (62, 5) 6 (37, 5)
Level of Education	
Diploma B.A M.A	1 (6, 25) 11 (68, 75) 4 (25)
Department/ Specialty	
Emergency CCU/ICU Neonatal Intensive Care Unit (NICU) Surgeical ward Other* (Dialysis and Gynaecology)	4 (25) 5 (31, 25) 2 (12, 5) 3 (18, 75) 2 (12, 5)
Years of work experience	
Less than 5 5–10 11–15 More than 15	0 (0) 6 (37, 5) 3 (18, 75) 7 (43, 75)
Specialty	
Head nurse/Supervisor Nurse Practical nurse	3 (18, 75) 12 (75) 1 (6, 25)
Previous experience attending disasters	
Yes No	7 (43, 75) 9 (56, 25)
Attending specialized disaster-related courses	
Yes No	6 (37, 5) 10 (62, 5)
Time to send nurses to earthquake-affected areas	
Within the first 24h after the earthquake 24–48 h after the earthquake	14 (87, 5) 2 (12, 5)
Duration of stay in earthquake-affected areas	
3–5 days 6–10 days	3 (18, 75) 13 (81, 25)

Data collection continued until reaching data saturation. Saturation is a characteristic that is closely related to the number of participants. Data saturation means that no new data or information is obtained by continuing data collection [19]. Participants were selected from different hospitals affiliated with Tehran University of Medical Sciences to ensure maximum variation. The exclusion criterion included unwillingness to participate in the study.

### Data collection

To collect data, the participants were present at their workplaces with prior coordination and appointment and conducted individual interviews with them in a calm environment. Semi-structured interviews were started using simple and general topics and progressed to specific questions. Initially, two unstructured interviews were conducted to determine the main line of the interview and complete the questions of the interview guide. To describe the participants, their demographic information was recorded. Individual interviews aimed to answer the following research questions:

Why did you choose to provide aid services to Kermanshah’s earthquake-affected areas? Can you describe a particularly positive or negative experience you had while providing aid services in Kermanshah? What were some of the challenges you faced when providing health and medical services in the Kermanshah earthquake-affected region? Can you provide specific examples?; How did you go about addressing these challenges? Can you describe the solutions you used?; In your opinion, what are the most pressing needs of nurses during the earthquake response phase?

All interviews were audio-recorded with the participant’s permission. Also, to ensure the accuracy of the data and obtain more comprehensive information, the researcher recorded all observations made during the interviews. These observational notes were then utilized as a data source in the data analysis process.

### Data analysis

Data analysis and data collection proceeded concurrently. Following the qualitative content analysis approach proposed by Graneheim & Lundman (2004) [20], recorded interviews were listened to multiple times, transcribed verbatim, and supplemented with notes. The transcripts underwent several readings to grasp the interview content. To analyze the interviews, meaning units were extracted from the transcripts and coded separately. The primary codes were then compared, and similar codes were organized into subcategories. Continuous comparison of subcategories based on suitability and similarity led to their placement within main categories representing the research’s main themes. To validate the codes, the transcript was revisited multiple times. Content analysis was manually performed on the Persian data before translation.

### Trustworthiness

Four criteria of credibility, dependability, transferability, and confirmability have been proposed to evaluate qualitative research [21].

Credibility, crucial for data acceptability, was reinforced through ten months of dedicated data collection and analysis, fostering long-term engagement with participants. The interviewer’s nursing involvement enriched data through friendly communication. Maximizing participant variation and conducting interviews at various times and places heightened research result reliability. Two qualitative study experts supervised and reviewed all interview transcripts and data analysis stages, and their additional comments were used. After the transcription, coding, and the emergence of the initial categories, the interviews were given to several participants, and they were allowed to give submit feedback. Participants approved all the items except for a few items, which were modified based on their comments.

Dependability refers to data stability and reliability under the same conditions and over time. One way to assess reliability is through external monitoring. For this purpose, an individual with a Ph.D. in nursing disaster and emergency health was used as an external monitor to ensure a consistent perception of the data.

Transferability in qualitative research refers to how well the findings of a study can be applied to other contexts or populations. To enhance transferability in this study, the researcher used several strategies such as simultaneous data collection and analysis, ensuring coherence between research questions and methodology, comparing results to those of other studies, step-by-step reporting of each research stage, and selecting a diverse range of participants. This approach ensured the findings were relevant to a broader audience beyond the specific study setting.

Confirmability refers to accuracy in all research stages, and the research method’s transparency can confirm the data. The researcher tried to document all stages of the research in detail so that readers could adapt it to their desired context and use it. The details of data collection and analysis, transcription, as well as the researcher’s thoughts and interpretations were recorded and preserved.

## Results

A total of 16 people participated in the present study, nine of whom were women (56.25%) and seven were men (43.75%). The majority of participants were married (62.5%), 35–44 years old (50%), had a bachelor’s degree (68.75%), and had no previous experience of being in disasters (56.25%). The participants were mainly from ICUs and emergency departments (68.75%). All participants had more than 5 years of work experience and were dispatched to the earthquake-affected areas within the first 48 h after the disaster. The majority stayed there for 6–10 days (Table 1). Interviews lasted 45–60 min (average duration: 52 min) and were conducted in Persian.

The analysis of the interviews categorized nurses’ experiences, which were dispatched during the Kermanshah earthquake into three main categories and 12 subcategories (Table 2).

**Table 2:** Results of data analysis

Categories	Subcategories	Codes
**Individual experiences**	satisfaction	The sense of pleasure / Mental refreshment when helping others Gaining a good feeling when talking with injured and needy people Gaining a good feeling because of effective group work
	Professional experiences	Using the facilities in the best way and creativity and innovation to compensate for the lack of equipment Learning to resist against problems/Increasing resilience and learning patience and perseverance
	Physical and mental experiences	Fatigue due to continuous work/Ignoring physical conditions at work Psychological problems due to working under disaster conditions/Bad mental conditions/Hard psychological conditions of working with injured children Anxiety and worry about future problems and dangers/Fear and panic caused by aftershocks Mental conflict and worry about the family situation Neglect of psychological issues of the disaster medical assistance teams (DMAT) after returning/Not receiving feedback after returning
**Operational experiences**	Command, leadership, and supervision	Lack of unit management and clear command Weak supervision and control over the process of delivering services
	Coordination	Poorly coordinated dispatch (delayed flights - return of several medical forces from the airport) Poorly coordinated dispatch to the affected areas Uncoordinated dispatch of medical forces to the earthquake affected area
	Organizing human resources	Giving no time off work to tired local forces in some areas and use of dispatched nurses as extra forces Lack of proper management in the use of forces/ Failure to use all the capacity and capabilities of the dispatched forces The feeling of having extra forces/ Feeling inefficient in some situations
	logistic	An increase in the number of medical staff in some areas and problems in supporting them/ Providing no support for nurses in some areas (resting in cold tents, risk of theft of portable equipment) Inappropriate clothing, etiquette, and organization for nurses
	Protocols and instructions	Absence of clear guidelines regarding the job description No description of specific duties
	Training and empowerment	Attendance of trained teams in disaster-affected areas Pre-disaster training Holding periodic maneuvers
	Continuation of service delivery after the response phase	Continuation of post-earthquake services by nurses Post-earthquake empowerment and rehabilitation of affected people
**Social and cultural experiences**	Social participation	People’s participation in collecting bodies and identifying the injured Availability of people affected during the war and using their experiences
	Cultural experiences	Using local forces/ Paying attention to the cultural issues of the region Facilitating people’s communication by employing medical forces of the same language

### Individual experiences

This main category represents the individual experiences of nurses who attended and provided aid to the earthquake victims of Kermanshah and includes:

#### Satisfaction

All participating nurses reported experiencing feelings of satisfaction while attending and providing aid services during the Kermanshah earthquake. They experienced a sense of pleasure, mental refreshment, and a positive feeling toward effective teamwork. Participants No. 9 and 1 specifically reported the following experiences:

*“I felt mentally refreshed because I was helping the earthquake victims.”*

*“There was a special intimacy between the team members. I felt satisfied when we were doing things as a group and has good cooperation to solve a problem.”*

#### Professional experiences

During their attendance and provision of aid services in the earthquake-affected areas, the participating nurses gained new professional experiences. They had to use creativity and innovation to compensate for the shortage of equipment, and they learned to be resilient and patient under the difficult conditions of the disaster. Participants No. 11 and 15 specifically reported the following experiences:

*“I learned to make the most of minimal facilities and resources and to be thrifty in my approach. In the face of equipment shortages, we had to be creative. For example, I had to make splints from wooden boards.”*

*“Observing the conditions of the earthquake-affected people, their patience and tolerance despite losing everything, and working under such difficult circumstances made me more mature and taught me to be more patient, resistant, and resilient.”*

#### Physical and mental experiences

One of the personal experiences of nurses is their physical and mental experiences under disaster conditions; they stated that they have ignored their physical conditions, worked around the clock, and felt fatigue. In this regard, Participant No. 7 said:

*“Due to the huge workload, I felt very tired, and I had not eaten anything at all, and due to my weakness and low blood pressure, my colleagues hooked me up to IV.”*

They also talked about the difficult psychological conditions of nurses working under disaster conditions, such as the difficult psychological conditions when working with injured and afflicted people, fear and panic caused by aftershocks, mental conflict with the situation of their family members, and ignorance of their psychological issues after returning. In this regard, Participants No. 8 and 12 said:

*“It was very difficult for me to see all the suffering of injured people. Seeing injured children who had lost their loved ones was very difficult. I was tormented by seeing all this, and I cried in private.”*

*“For a long time after returning, I was still affected by the events there, cried remembering the memories, and needed mental recovery, but nobody paid attention to us.”*

### Operational experiences

This main category pertains to the operational experiences that the participating nurses encountered during the disaster response and includes:

#### Command, leadership, and supervision

The nurses reported a lack of unit management and clear command as a weakness during the disaster response. Upon arriving at the earthquake area, they found the situation confusing and were unsure who to report to or what duties to perform. Participants No. 1 and 5 noted the following experiences:

*“The earthquake area was very crowded and chaotic upon our arrival. We were uncertain about who to report to andour responsibilities.”*

*“Due to the absence of unified management, there was sometimes inadequate supervision and control over the working process. Nurses could not always ensure that duties were being performed correctly.”*

#### Coordination

In the response process, the lack of coordination in the dispatch process to the lack of coordination with the local authorities was one of the experiences of the participating nurses. In this regard, Participants No. 2 and 11 said:

*“Even though we were asked to arrive at the place for deployment immediately, we faced a delayed flight for a few hours. On the other hand, many DMATs had arrived at the airport, and some were asked to return due to poor coordination.”*

*“The manager of the medical center located in the earthquake-affected area was not informed of our dispatch. When we arrived, he said that we were facing an oversupply of DMATs here and we don’t need you, and you should come back.”*

#### Organizing human resources

Poor organization of DMATs in the response phase was also one of the negative experiences of nurses in the operational field. In this regard, Participant No. 8 said:

*“Some of the native nurses were very tired after hours of non-stop work, but they were still active, and although we arrived there recently, we could not play an effective role, and our full capacity was not used well.”*

#### Logistic

Nurses also experienced poor logistic operations of the DMATs in the response phase in some earthquake-affected areas. In this regard, Participant No. 3 said:

*“In some earthquake-affected areas, we had to rest in a tent. There were no suitable and sufficient heating devices, and the weather was very cold. There was no clear and safe place to put our personal belongings.”*

Another major logistical challenge was related to the inappropriate distribution of equipment. In this regard, Participant No. 7 said:

*“The equipment and resources were not adequately distributed, and the abundant aid sent, which was substantial, was not well-distributed in a manner that left a significantamount of resources unused in certain areas”.*

#### Protocols and instructions

The nurses mentioned the lack of clear protocols and instructions as one of the operational weaknesses. In this regard, Participant No. 13 said:

*“There was no clear job description so that people could do it based on their expertise, which led to a decline in efficiency. Job speed and accuracy can increase by knowing about each person’s job description.”*

#### Training and empowerment

Training and empowerment, the presence of trained teams in the disasters, pre-disaster training, and periodical maneuvers were important from the perspective of the participating nurses. In this regard, Participant No. 16 said:

*“The first thing I would say is that managers should dispatch trained nurses there than me, who have never attended a training course. Because disaster situations are special conditions. Make sure to hold a training course before a disaster occurs. For example, don’t let an earthquake occur, and then start training nurses. There must already be a group of trained nurses for such situations.”*

#### Continuation of service delivery after the response phase

This issue was also one of the operational concerns of nurses. In this regard, Participant No. 7, for example, stated:

*“Affected people should be reassured that we are thinking about them and they will not be forgotten even after this acute phase. Some people were worried that we would not contact them later. The provision of services should continue until all their problems are resolved, and administrators will do the necessary planning.”*

### Social and cultural experiences

This main category represents the social and cultural experiences of the nurses attending and provdingi aid services to Kermanshah’s earthquake-affected areas.

#### Social participation

The public role and participation during the response phase were also one of the positive points raised by the nurses. Nurses stated that people in some remote areas had sufficient capability and resilience to manage the situation, considering their previous experiences in the war. On this note, Participant No. 15 highlighted:

*“The people of some earthquake-affected areas cooperated well with the aid workers considering their previous experiences in war and the difficult living conditions in that area. They had great patience and tolerance in the face of problems. When we entered a remote village, the local people cooperated and pulled out the bodies from under the rubble. They also cooperated with the aid workers to identify and help the injured. People’s capability helps a lot in better disaster management.”*

#### Cultural experiences

Nurses also referred to the cultural affinity of the DMATs and the use of local DMATs to help facilitate communication between local DMATs and the people who share the same language. On this note, Participant No. 5 highlighted:

*“Some of the old men and old women spoke the region’s local language and did not understand Farsi. Therefore, it would be better to prioritize and employ nurses from neighboring cities and provinces, who have a closer cultural affinity to the region under this situation.”*

## Discussion

The study’s main findings encompass three categories and 12 subcategories: individual experiences (satisfaction, professional, and physical/psychological), operational experiences (command, leadership, coordination, organization, logistics, protocols, training, and continuous service provision), and social/cultural experiences (social participation and cultural encounters). Notably, nurses displayed high motivation, receiving calls from hospital managers or volunteering after being informed of the disaster the following morning.

In terms of individual experiences, nurses conveyed a profound sense of satisfaction derived from their involvement in providing aid services in earthquake-affected areas. They described experiencing mental rejuvenation and joy when assisting those in need, a sentiment consistent with findings from other studies. For instance, Nasrabadi et al. (2007) highlighted nurses’ happiness and satisfaction in their humanitarian efforts and caring for the injured [10]. Similarly, in Yang et al.‘s (2010) research, nurses found meaning in their assistance to earthquake-affected areas, emphasizing the value and significance of such experiences [22]. Additionally, this study identified that the mutual care and support among team members contributed to effective teamwork, aligning with the study’s emphasis on the satisfaction derived from collaborative efforts.

Participating nurses showed resourcefulness in using available facilities creatively amid equipment shortages. Their professional experiences in disaster conditions, such as multitasking and adapting to limited resources, resonate with findings from Yang et al. (2010) [22]. Nurses in this study discussed personal growth, resilience, and skill enhancement, aligning with similar observations in previous disaster response studies [8, 9, 22, 23].

Nurses in disaster situations experience notable physical and mental challenges, including fatigue and neglect of well-being due to continuous work [24]. Maintaining good physical and mental health is essential for nurses to effectively work in disaster conditions, emphasizing the need to address environmental factors [25]. The nurses described significant psychological challenges, such as anxiety and worry about future issues and dangers, heightened by fear from aftershocks. Mental conflicts related to family situations and a lack of awareness about their psychological well-being post-return were also prominent. These findings resonate with studies documenting mental and emotional stress among caregivers and those affected by disasters [26]. Nurses in disaster response teams often grapple with psychological distress exacerbated by inadequate management, communication mechanisms, and resources. A study reported a high incidence of acute stress disorder (ASD) symptoms among nurses during relief efforts, with some experiencing post-traumatic stress disorder (PTSD). Contributing factors included the severity of earthquake casualties, disconnection from headquarters, and safety-related fears during aftershocks [22]. The study highlighted anxiety and worry about future problems and dangers due to aftershock-related fear and panic, aligning with Pourvakhshoori et al.‘s findings (2017). The primary concern reported by nurses during disasters was the fear of aftershocks, reflecting significant fear and anxiety among participants. Similar to Porukhshui et al., the safety of nurses’ families emerged as the primary mental concern [9]. Australian aid workers emphasized the importance of communication with family members during disasters, suggesting predetermined channels and strategies to enhance their involvement [27]. Addressing challenges, such as rotating shifts, facilitating family information exchange, and supporting those struggling, can mitigate emotional impact without affecting medical services.Nurses should be aware of the short- and long-term effects of disasters, employing coping strategies and creating support networks for themselves and colleagues (12). Disaster nursing courses must incorporate strategies to manage psychological reactions for both survivors and nurses in traumatic situations [22].

Participants in our study highlighted challenges related to the lack of clear command, leadership, and supervision during disaster response. They expressed confusion in receiving commands from various sources, leading to inadequate performance monitoring. Emphasizing the importance of unified management, clear command, and intersectoral coordination, the nurses suggested that improving efficiency and proper disaster management requires coordination and communication among responders and stakeholders. This sentiment aligns with findings from Nasrabadi et al. (2007), who stressed the significance of teamwork and the role of a team leader, underlining the need for a leadership center and coordination to avoid duplicate efforts [10]. Similar concerns about non-cooperation among disaster medical assistance teams (DMAT) and insufficient leadership were reported by Li et al. (2017), highlighting challenges in coordinating resources and communicating with local healthcare services and other relief teams [8]. Bikmohammadi et al. (2015) also identified the absence of a practical disaster commanding system in the response phase among emergency department nurses [28]. To address these issues and enhance disaster response management, Waugh & Streib advocated for training courses focusing on coordination and communication among different organizations (29). Successful disaster management on a large scale involves multiple organizations, and effective coordination is crucial due to the extensive nature of disaster-related activities. The ultimate goal is to minimize subsequent damage and losses [30]. In Iran, there is a lack of a coherent plan and structure to address coordination challenges, contrasting with many developing countries that have established independent and codified structures [31].

Operational experiences of the nurses revealed challenges in organizing and supporting medical assistance teams in certain earthquake-affected areas, which they considered a weakness. The increase in staff numbers in some areas led to a perception of inefficiency and a burden on the existing resources under these circumstances. Furthermore, certain nurses highlighted a shortage of logistical resources, including inadequate tent accommodations and extremely cold weather conditions, leading to a sense of insecurity regarding the theft of personal belongings. Li et al.‘s study (2017) identified challenges related to the disorganization of volunteer disaster medical assistance teams (DMAT) [8]. Nurses in another study shared concerns about personal safety, family obligations, and insufficient provisions of food, water, and rest [9], aligning with the findings of the present study.

In their operational experiences, the nurses in this study highlighted the lack of a specific protocol and instructions, impacting their efficiency. They emphasized that unclear instructions regarding duties prevented them from utilizing the full capacity and capabilities of the workforce, a concern consistent with Nasrabadi et al.‘s study on nurses’ experiences in the Bam earthquake in Iran. That study underscored the necessity of preparing practical protocols as part of an initial plan in disaster management, identifying the absence of protocols as a challenge for nurses. It also noted increased efficiency and effectiveness when practical protocols were in place [10]. Addressing the role of nurses in providing services at different stages of disaster management is crucial. The International Council of Nurses, prioritizing nursing in disasters, has stressed the importance of nurses being prepared to deliver services at various stages, with clear instructions about their location and job description in advance [13]. To meet public expectations and guide nurses, the development of national disaster nursing guidelines and concepts is recommended [14].

This study highlights the crucial need for pre-disaster training and preparation, emphasizing the importance of training and empowering nurses to handle disaster conditions effectively. In line with the WHO’s global recommendations, it suggests that all countries prioritize national and local pre-disaster training efforts, irrespective of their disaster frequency [32]. This perspective is supported by various studies. For instance, Nasrabadi et al.‘s study suggests that nurses who periodically update their knowledge work more effectively in real situations, exhibiting greater confidence and making fewer mistakes [10]. Li et al.‘s study (2017) echoes this sentiment, with nurses highlighting the importance of planning for preparation, training, and practice, consistent with the experiences shared in the present study [8]. Additionally, research indicates that Simulation and practice in disaster nursing courses enhance realism, leading to improved learning outcomes. The incorporation of real-life scenarios into educational programs is considered crucial for innovative teaching and learning [22].

Continuous provision of services beyond the response phase emerged as another operational experience for nurses. Their role becomes increasingly crucial during the recovery and rehabilitation stages, aligning with the comprehensive care approach. Disaster recovery planning should prioritize long-term nursing services to address community needs effectively. Nurses, with their understanding of people’s needs and adaptability to provide a diverse range of services, play a pivotal role in restoring the community to its pre-disaster state [12]. The ICN Framework of Disaster Nursing Competencies highlights the importance of nurses’ knowledge in various stages of recovery, encompassing individual, family, and social long-term recovery care [7]. Developing nursing strategies for disaster survivors requires systematic planning and long-term studies. Education for professionals, families, friends of survivors, and the community about life after a disaster is equally essential [12].

Nurses highlighted positive social participation, drawing on people’s resilience from past experiences, particularly during years of war. Community-based disaster management (CBDM) emerged as a vital approach, fostering adaptive capacity and resilience throughout the disaster cycle. CBDM involves active participation in decision-making, planning, and implementation to prevent disasters or restore communities, utilizing local and global resources. Recognizing diverse participation opportunities is crucial for effective community engagement [33].

Nurses highlighted cultural issues, emphasizing the importance of cultural affinity within Disaster Medical Assistance Teams (DMATs). They identified challenges in communication due to language barriers with the earthquake-affected people who predominantlyspoke Kurdish. The nurses recommended the involvement of native nurses to enhance effective communication, echoing findings from Yang et al. (2010) regarding the benefits of collaboration between dispatched and local community nurses for improved response effectiveness and stress reduction [22]. Effective communication is crucial for patient safety, group dynamics, and overall satisfaction in healthcare settings [34]. In this study, nurses emphasized the importance of effective communication, particularly with native people, proposing the involvement of local nurses who can better understand and navigate the communication challenges.

Limitations include focusing solely on the response phase of the Kermanshah earthquake, potentially missing long-term experiences. The study’s applicability is constrained to nurses from Tehran University of Medical Sciences, limiting generalizability. Despite these limitations, the research offers valuable insights into nurses’ experiences during the earthquake, suggesting future studies explore long-term effects and perspectives of various healthcare professionals and emergency responders, as well as broader contextual factors influencing disaster response effectiveness.

## Conclusion

In conclusion, this study sheds light on nurses’ experiences providing healthcare during the Kermanshah earthquake. The findings highlight the importance of disaster nursing and nurses’ key role in disaster prevention, preparedness, response, and recovery. Specifically, the study identifies several important factors that influenced nurses’ experiences during the response phase of the disaster, including the need for effective teamwork, emotional support, and clear command and coordination. Based on these findings, future research could explore ways to enhance disaster nursing education and training programs to better prepare nurses for providing healthcare during disasters, particularly in promoting effective teamwork and addressing the emotional challenges of disaster nursing. Additionally, policymakers and healthcare organizations could use the findings to inform the development of disaster response plans that better address the needs of nurses and promote effective communication and coordination among healthcare providers and other emergency responders. The study underscores the importance of continued research and practice in disaster nursing to improve the response to disasters and promote better health outcomes for affected individuals and communities.

### Electronic supplementary material

Below is the link to the electronic supplementary material.

Supplementary Material 1

## Data Availability

The datasets used and/or analysed during the current study are available from the corresponding author on reasonable request.
