# Supplementary material for: Nurses’ experiences of providing medical services during the Kermanshah earthquake in Iran: a qualitative study
Source: BMC Emerg Med. 2024 Jan 7;24:4. doi: 10.1186/s12873-023-00920-9 (PMC10773068; doi:10.1186/s12873-023-00920-9)
Supplement: Supplementary file 1 — Supplementary Material 1 [file 12873_2023_920_MOESM1_ESM.docx]

**Qualitative Interview Guideline in Nurses' Experiences of Providing Medical Services During the Kermanshah Earthquake in Iran: A Qualitative Study**

**Introduction:**

Begin by introducing yourself and your role in providing aid services in Kermanshah.

Explain the purpose of the interview and assure confidentiality.

**Questions:**

**Question 1:** Why did you choose to provide aid services to Kermanshah's earthquake-affected areas?

**Question 2:** Can you describe a particularly positive or negative experience you had while providing aid services in Kermanshah?

**Question 3:** What were some of the challenges you faced when providing health and medical services in the Kermanshah earthquake-affected region? Can you provide specific examples?

**Question 4:** How did you go about addressing these challenges? Can you describe the solutions you used?

**Question 5:** In your opinion, what are the most pressing needs of nurses during the earthquake response phase?
